# Movement Behavior and Host Location Ability of *Corythucha ciliata*

**DOI:** 10.1371/journal.pone.0152205

**Published:** 2016-03-28

**Authors:** Haiwei Wu, Huanxiu Liu

**Affiliations:** Shandong Academy of Forestry, Jinan, Shandong Province, China; East Carolina University, UNITED STATES

## Abstract

Insect movement behavior is highly important in entomological population ecology, behavioral ecology and conservation, and in invasion ecology. In this study, we used an exotic lace bug (*Corythucha ciliata*) as a model organism to address the hypothesis that an insect species invading a new area has a high host location ability and rapid mobility by which it can be successfully carried to a new habitat. To test this hypothesis, three movement parameters (speed, duration and distance) of *C*. *ciliata* were assessed using laboratory and field observations. We found that 5^th^-instar nymphs of *C*. *ciliata* could move as far as 750 cm throughout their lifespan and that they moved an average of 0.038 m/min during the first 15 minutes after release, which was significantly farther than that of other instars. Of the tested nymphs, 21.85% could locate their host trees; of adults released 20 m from hosts, 11% reached the host trees on the first day, with an average flight distance of 22.14 m and a maximum flight distance of 27 m. The results of this study partly explain the mechanism of rapid diffusion. These results are also important for predicting population spread, improving eradication surveys, and managing future introductions of *C*. *ciliata*.

## Introduction

Dispersal (i.e., the spatial redistribution of populations) is one of the most significant components of entomological population ecology, behavioral ecology and conservation [1–4]. Because dispersal is (in part) random movement of individuals, which underlies the redistribution in space of a population [5–8], there is a close relationship between movement (i.e., the spatial behavior of individuals) and dispersal. Movement of insects is of central importance for them to avoid natural enemies [9], search for food [10,11], secure mates [12], and find suitable habitats [12,13].

In insect population management, movement of individuals strongly influences the area over which management practices should be applied either to increase (conservation) or decrease (pest control) the abundance of a species of interest [14]. Information on movement parameters (speed, duration, and orientation) of insect pests is essential for their control. Meanwhile, understanding the movement behavior of natural enemies is also helpful for developing effective release techniques and for predicting the efficacy of releases [15–17].

Flying (by adults) and crawling (by larvae) are the two main methods of active movement of insects throughout their life history. Studies on the dispersal ability of adult insects abound in the literature [5,15–26]. In contrast, few studies have measured or modeled the parameters of insect movement in larval populations for most insects, partly due to the absence of effective attractants for larvae [27].

We focus on movement behavior in the sycamore lace bug, *Corythucha ciliata* (Say) (Hemiptera: Tingidae). This pest is native to North America but has successfully invaded many countries in Europe [28], Australia [29], and Asia [30–32]. In China, *C*. *ciliata* was initially discovered in 2006 in Wuhan, Hubei Province [33]. To date, infestations have been reported in Shanghai, Zhejiang, Anhui, Jiangsu, Guizhou, Sichuan, Chongqing, Hunan, Hubei, Henan, Shandong and Beijing [34]. *C*. *ciliata* is a specialist herbivore with a narrow host range within the genus *Platanus*. It has five generations per year in the above-mentioned distribution areas in China, with five immature instars per generation [35]. There are many other bioecological characteristics supporting the rapid spread of *C*. *ciliata*, such as high fecundity [36], high thermal tolerance [37,38], robust cold tolerance [39] and strong starvation resistance [40]. We hypothesize that this species may also have a high movement speed and a high capacity for host location, both of which play important roles in its rapid spread.

In the present study, we conducted laboratory and field observations to assess three movement parameters (speed, duration and distance) and the capacity of host location of *C*. *ciliata* under conditions of no food and no water. The study addressed the following specific questions: (1) How fast could *C*. *ciliata* nymphs move? (2) How far could nymphs at different ages move before they died? (3) What is the rate of colonization of new hosts by *C*. *ciliata* nymphs? (4) Could *C*. *ciliata* adults find their hosts within a certain distance? The findings of this study improve the understanding of the dispersal capacity of this new pest, which is important for predicting population spread, improving eradication surveys, and managing future introductions.

## Materials and Methods

### Insects

Tested *C*. *ciliata* adults and nymphs were collected from London plane trees (*Platanus acerifolia* (Ait.) Willd.) at a nursery of Zhongjihuajing Garden Co., Ltd, in an experimental field of the Shandong Academy of Forestry and in the Licheng District of Ji'nan City (36.846°N, 117.32°E), which has had an infestation of *C*. *ciliata* for two years. All the *P*. *acerifolia* trees and insects were kept pesticide-free in the field.

### Test of movement speed of *C*. *ciliata* nymphs

**Fig 1:**
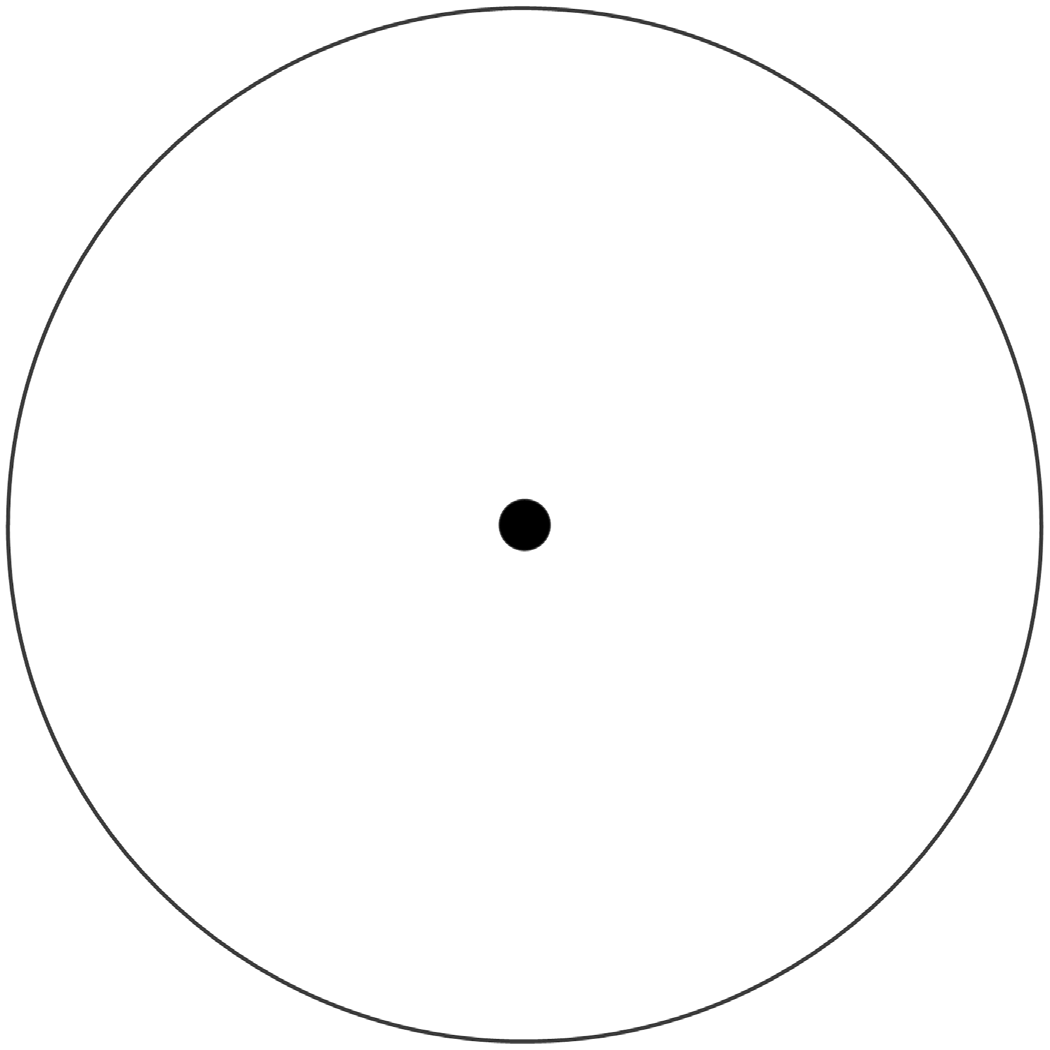
Model for testing the movement speed of *C*. *ciliata* nymphs. The radius of the circle is 0.4m, Solid dot represents release point of nymphs.

Based on the test methods of Bach [41], experiments on nymphal movement speed were carried out in flat soil ground devoid of vegetation from 1 August to 15 August, 2015. Each experiment was started at 8:00 a.m and involved drawing a circle on the ground to create an observation arena 0.8 m in diameter (The diameter of the circle was determined based on a prepared test of crawling distance of first-instar nymphs of *C*. *ciliata* on paper). Five nymphs, one of each instar, was placed in the center of the arena and observed until it crossed the circumference of the circle. When this occurred, their crawling time from center to the circumference of the circle was recorded. Any nymph that did not cross the circumference of the circle after 3 hours was discarded. Thirty individuals of each instar were tested for the treatment. Each experiment was conducted under sunny and warm weather conditions. The average maximum temperature was 28.03°C and minimum temperature 26.69°C. The average relative humidity was 81.79%. Wind was also mild during the experiments, averaging 2.28 m/s (Weather data were recorded by a weather station at Xinglong Town). A new observation arena was used for each experiment to avoid possible chemical cues from previous nymphs. Fig 1 showed a schematic view of the above experimental design.

### Determination of crawling distance of *C*. *ciliata* nymphs

**Fig 2:**
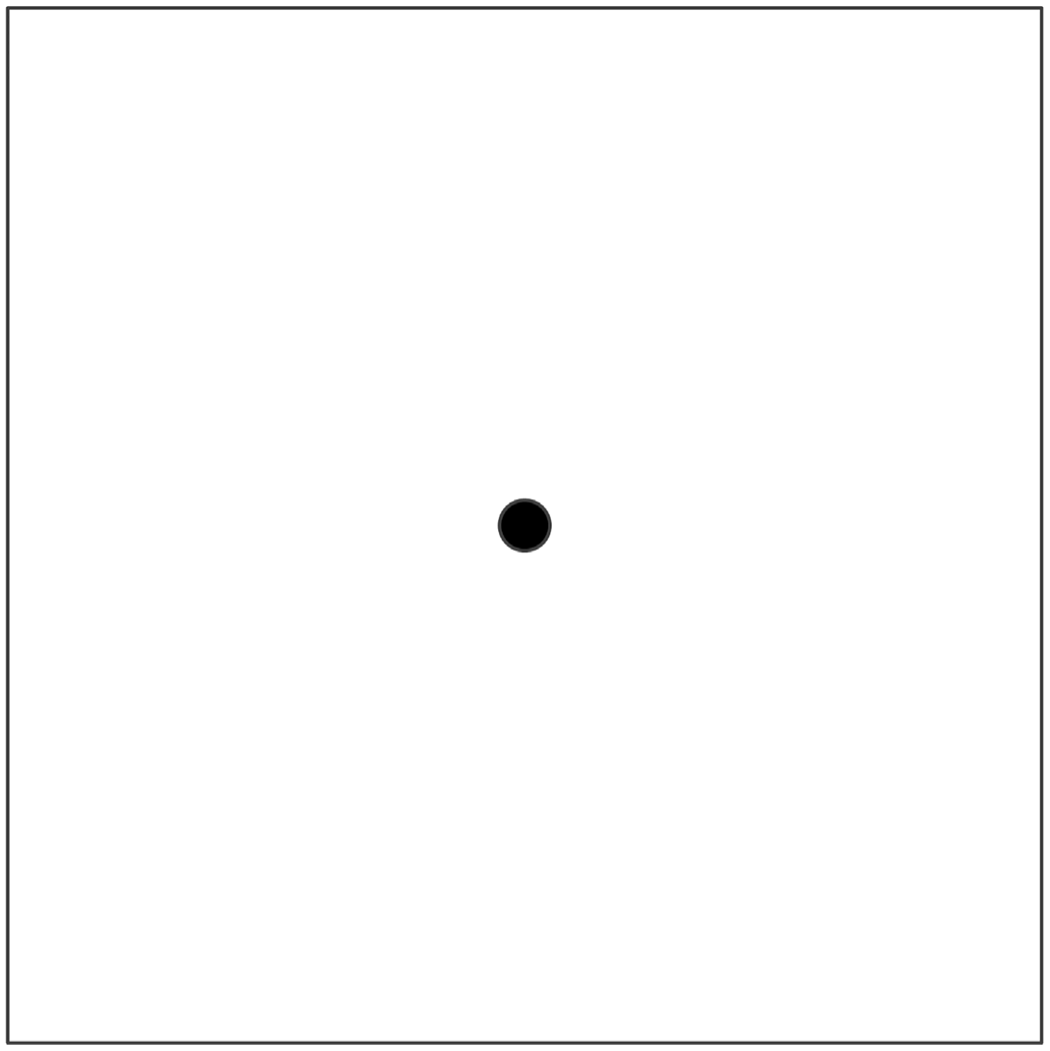
Model for testing the crawling distance of *C*. *ciliata* nymphs. Side length of the square is 4m, Solid dot represents release point of nymphs.

Experiments were carried out on square pieces of white paper (4 m×4 m) in the laboratory from 5 July to 15 July, 2015. Five nymphs, one of each instar, were placed in the center of the paper, and their respective movement tracks were drawn with marker throughout the experiment. Observation continued until the nymphs were dead. When each nymph died, the time was recorded, and the crawling distance was measured with a measuring tape. A nymph was considered dead if none of its appendages moved after all appendages were touched with a brush [33]. Thirty individuals of each instar were tested for the treatment. Each experiment was conducted on a new paper to avoid possible chemical cues from previous nymphs. The temperature was maintained in the laboratory at 26 ± 0.5°C and the relative humidity was 70 ± 5%. Fig 2 showed a schematic view of the above experimental design.

### Test of host location of *C*. *ciliata* nymphs

**Fig 3:**
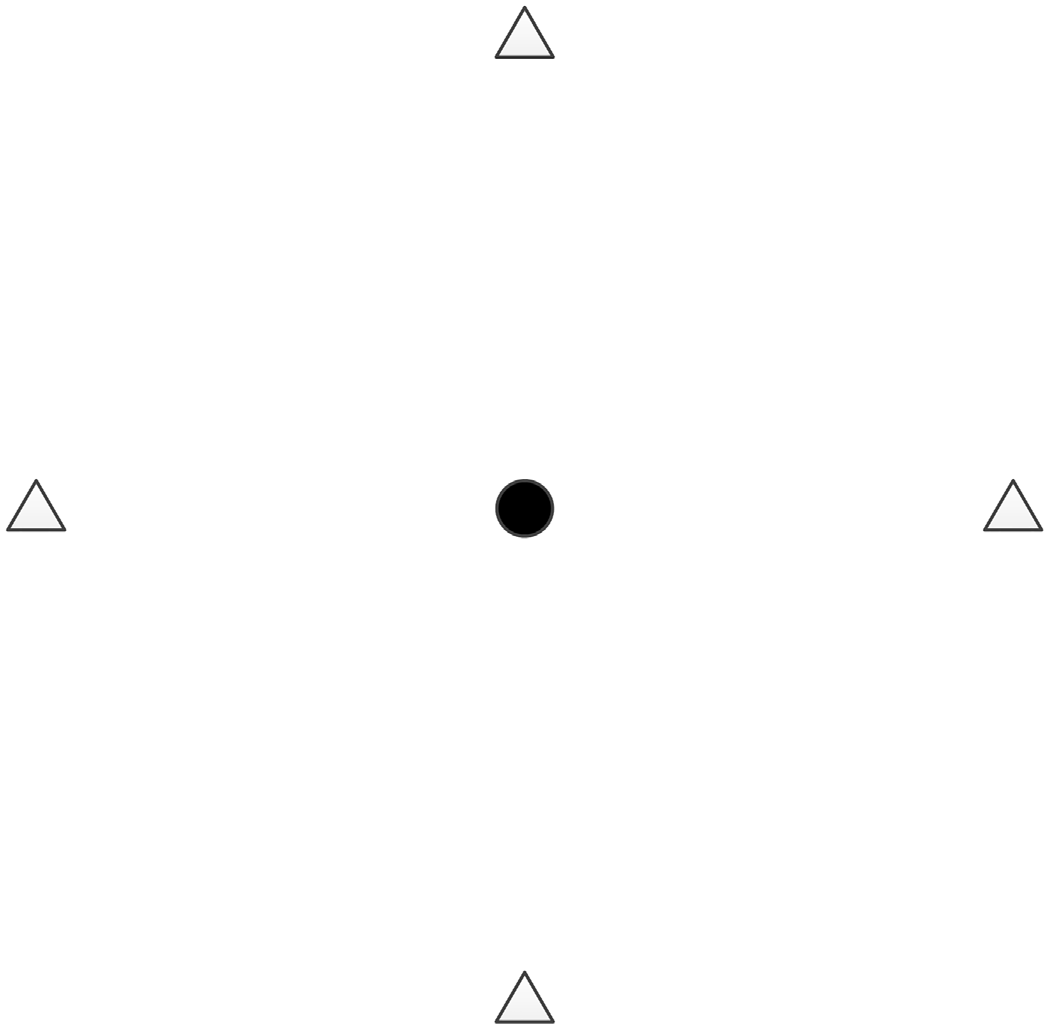
Model for testing the host location of *C*. *ciliata* nymphs. Solid dot represents release point of nymphs; triangles represent position of the host. The distance from solid dot to the four triangles was all 40cm.

Experiments were carried out in a flat soil ground devoid of vegetation in Xinglong Town, Linyi County, Dezhou City (37.116°N, 116.745°E) from 16 August to 19 August, 2015. Before each experiment, four water-filled bottles were buried upright underground at 0.4 m north, south, east, and west from a central point. Then, four *P*. *acerifolia* branches with 3–5 leaves each were inserted into each bottle; each branch reached approximately 0.5 m above the ground, and there were no gaps in the surrounding soil. Thirty nymphs of each instar were placed in the center of the arena every morning at 8:00 a.m. The number of different ages of *C*. *ciliata* nymphs climbing up the branches was recorded over 24 hours, which was the longest lifespan for *C*. *ciliata* nymphs without food and water [35]. *Populus canadensis* branches of the same thickness and height were used as controls. Three replicates were evaluated for each treatment. During all the experiments the maximum temperature was 32.80°C and minimum temperature 23.20°C. The average relative humidity was 78.26%. The average wind speed was 2.11 m/s (Weather data were recorded by a weather station at Xinglong Town). Fig 3 showed a schematic view of the above experimental design.

### Test of host location of *C*. *ciliata* adults

**Fig 4:**
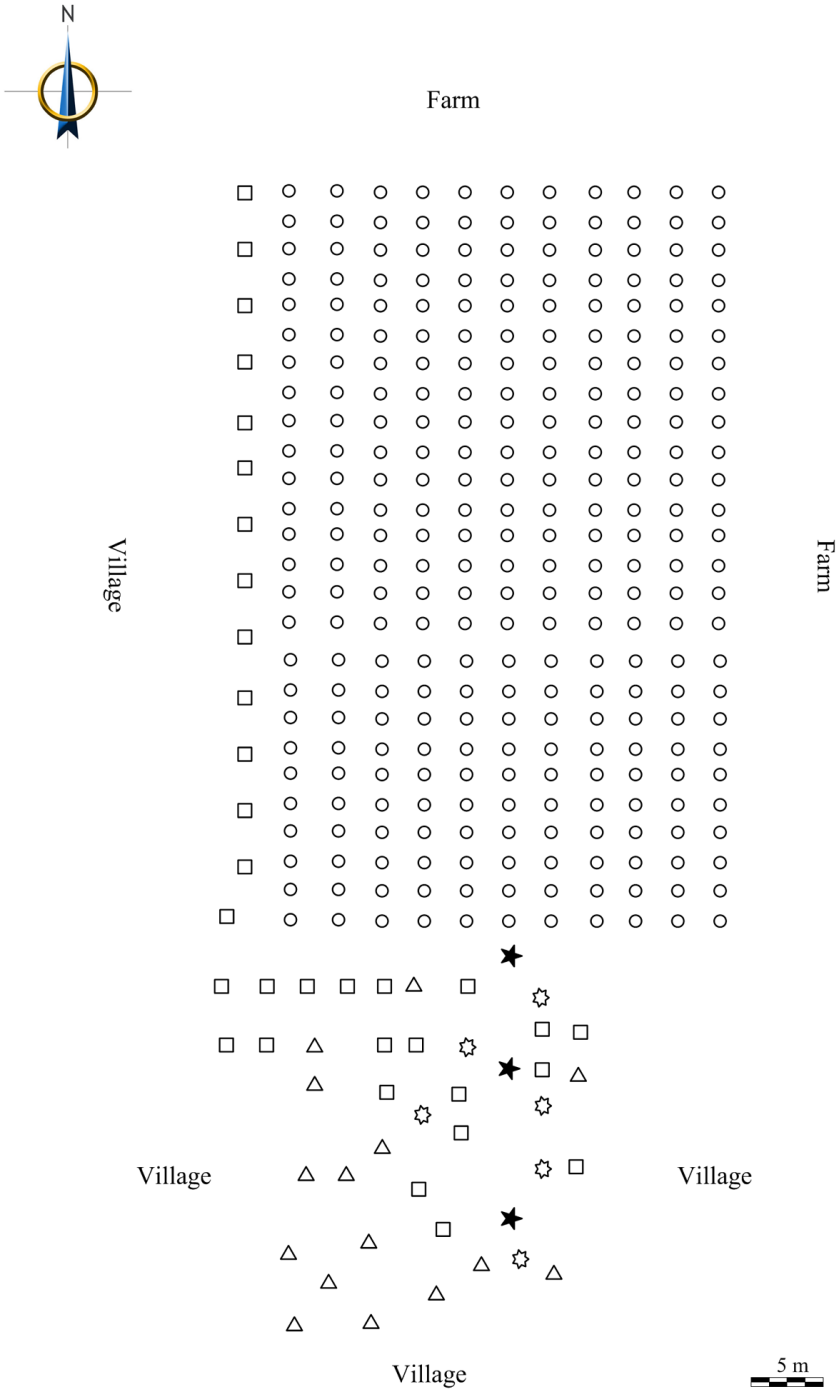
Map of study area. Circles represent sycamores, triangles represent poplars, squares represent elms, seven horns stars represent jujubes, and pentagram represent release points.

The experiments were performed in Xinglong Town, Linyi County, Dezhou City (37.116°N, 116.745°E); the collected *P*. *acerifolia* trees (height 1.0–1.5 m) were planted in a 30 m×50 m field parcel (11 tree rows×26 trees) in November 2014. There were no *Platanus* trees or *C*. *ciliata* insects nearby, only small numbers of jujube, poplar and elm trees. On 19 August 2015, 3–5 leaves bearing approximately 100 adults (regardless of sex) were placed at a release point that was 2 m away from the middle tree of the southern edge of the forest plot. All *Platanus* trees were visually surveyed at the second day after the initial release; the number of *C*. *ciliata* in each tree was enumerated, and the distance from the release point was measured with a measuring tape. The same process was conducted on 29 August 2015 and 8 September, with release distances of 10 m and 20 m, respectively. All of the adults that were found in *Platanus* trees were killed before additional insects were released. The interval of 10 days also ensured that there would be no living adults on *Platanus* trees because adult *C*. *ciliata* have a lifespan of 4–10 days in the field [42]. The release distances of 10 m and 20 m were based on the previous tests. Weather data were recorded by a weather station at Xinglong Town; weather during all experiments was sunny and warm, with an average temperature of 24.97±0.68°C. Wind was also mild during the experiments, averaging <4.80±0.12 m/s, and wind direction was predominantly from the southwest. Given the *C*. *ciliata* was not new invasive pest in Xinglong town and our test field was relatively closed and isolated, no host plants exist around. We signed a cooperation agreement with Xinglong Town People's government to kill all the *C*. *ciliata* in our test field after trial. A map of the study area is shown in Fig 4.

### Statistical analyses

The results are presented as the mean values ± SE, and all the means of movement parameters were compared among the different ages of nymphs using one-way analysis of variance (ANOVA). The difference in the rate at which nymphs climbed up the branches (RNCB) for host trees and non-host trees was analyzed using the *t-test* for independent samples. Statistical analyses were performed with SPSS (version 15.0, 2006, SPSS, Inc., Chicago, IL). Prior to ANOVA, all data were checked for normality and equal variance. When treatment effects were significant (P <0.05), the means were compared using Tukey's test. Moving speed, distance, times and RNCB were log-transformed, and the distance per time was sine-transformed prior to statistical analysis.

## Results

### Moving speed, distance and times of *C*. *ciliata* nymphs

The different ages of *C*. *ciliata* nymphs tested showed large differences in moving speeds (moving speed: *F*_*3*,*83*_ = 39.15, *P*<0.001; crawling distance: *F*_*4*,*99*_ = 21.93, *P*<0.001; crawling times: *F*_*4*,*99*_ = 5.25, *P*<0.001), but there were no significant differences in crawling distance per unit time (*F*_*4*,*99*_ = 1.62, *P*>0.1). Both the moving speed and the crawling distance increased with the increase of the age of the *C*. *ciliata* nymphs. The 5^th^-instar nymphs moved significantly faster than other instars, and 2^nd^-instar nymphs moved significantly more slowly than 3^rd^-instar and 4^th^-instar nymphs, between which there was no significant difference. The moving speeds of nymphs from the 2^nd^, 3^rd^, 4^th^, and 5^th^ instars were as shown in Table 1; 1^st^-instar nymphs was too small to be surveyed in this test. The crawling distance of the 1^st^-instar nymphs was significantly shorter than those of the other four instars throughout the life of each stage, averaging 41 cm. The mean crawling distance of 90.24 cm for 2^nd^-instar nymphs was also significantly shorter than those of the 3^rd^-instar, 4^th^-instar and 5^th^-instar nymphs, but there was no significant difference between these three instars (3^rd^ instar: 165.21 cm; 4^th^ instar: 174.72 cm; and 5^th^ instar: 291.35 cm). One 5^th^-instar nymph moved 750 cm, which was the longest distance of all the tested nymphs. The tested 1^st^-instar nymphs crawled for approximately 8.75 minutes throughout their lifespan, which was significantly less than for the other four instars. The crawling times of 2^nd^ to 5^th^ instar nymphs were not significantly different at 16.89, 19.20, 18.78, and 22.70 minutes, respectively (Table 1).

**Table 1:** Moving Speed, Distance and Times of *C*. *ciliata* Nymphs.

Instars	Number of nymphs	Moving speed (m/min)	Distance (cm)	Time (min)	Distance/time
1st	24	- -	41.00±6.79 c	8.75±1.27 b	5.42±0.94 a
2nd	28	0.008±0.001 c	90.24±11.03 b	16.89±2.22 a	5.92±0.56 a
3rd	20	0.017±0.001 b	165.21±19.44 a	19.20±3.37 a	10.28±0.97 a
4th	26	0.020±0.001 b	174.72±19.57 a	18.78±2.49 a	10.92±0.76 a
5th	23	0.038±0.003 a	291.35±43.26 a	22.70±3.56 a	13.79±1.26 a

* Means within the same column followed by different letters(a, b and c) are significantly different (Tukey’s test: P < 0.05).

### Host location of *C*. *ciliata* nymphs

**Fig 5:**
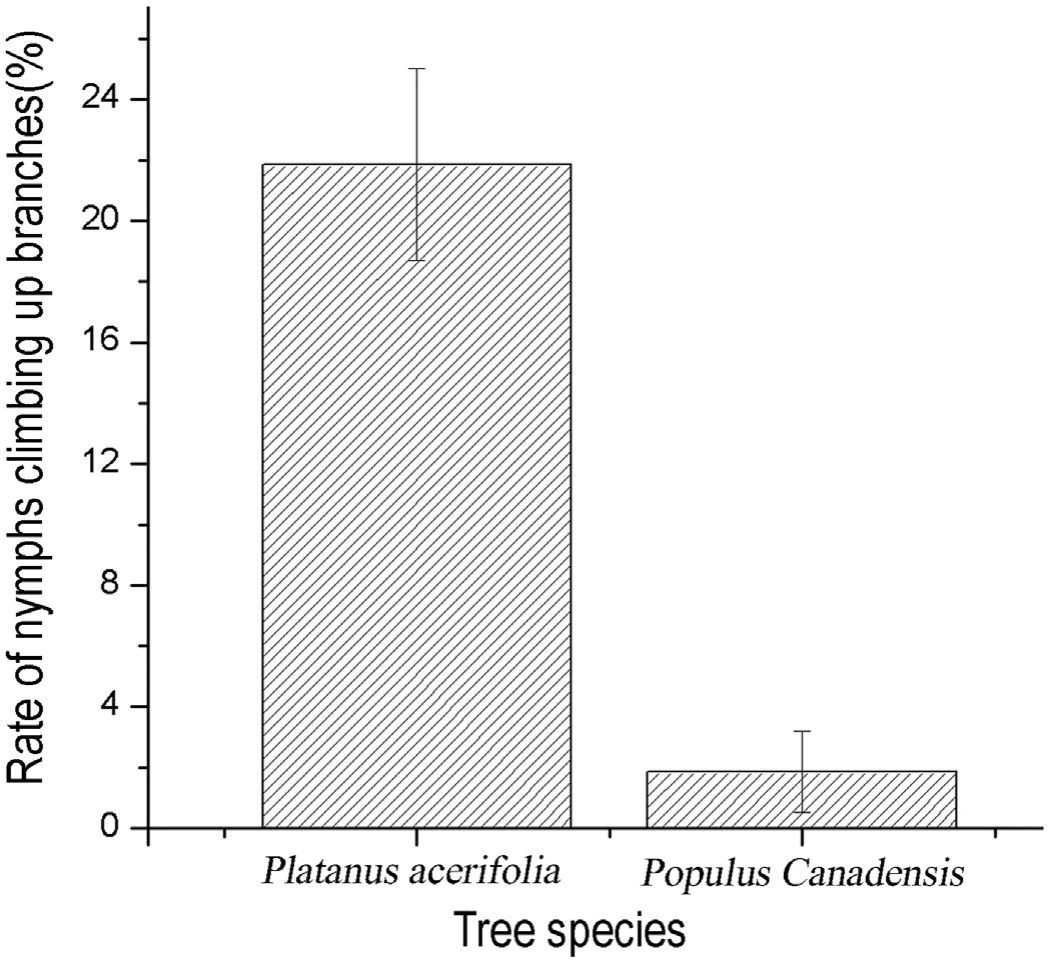
Rates of Nymphs Climbing Branches of Different Tree Species.

**Fig 6:**
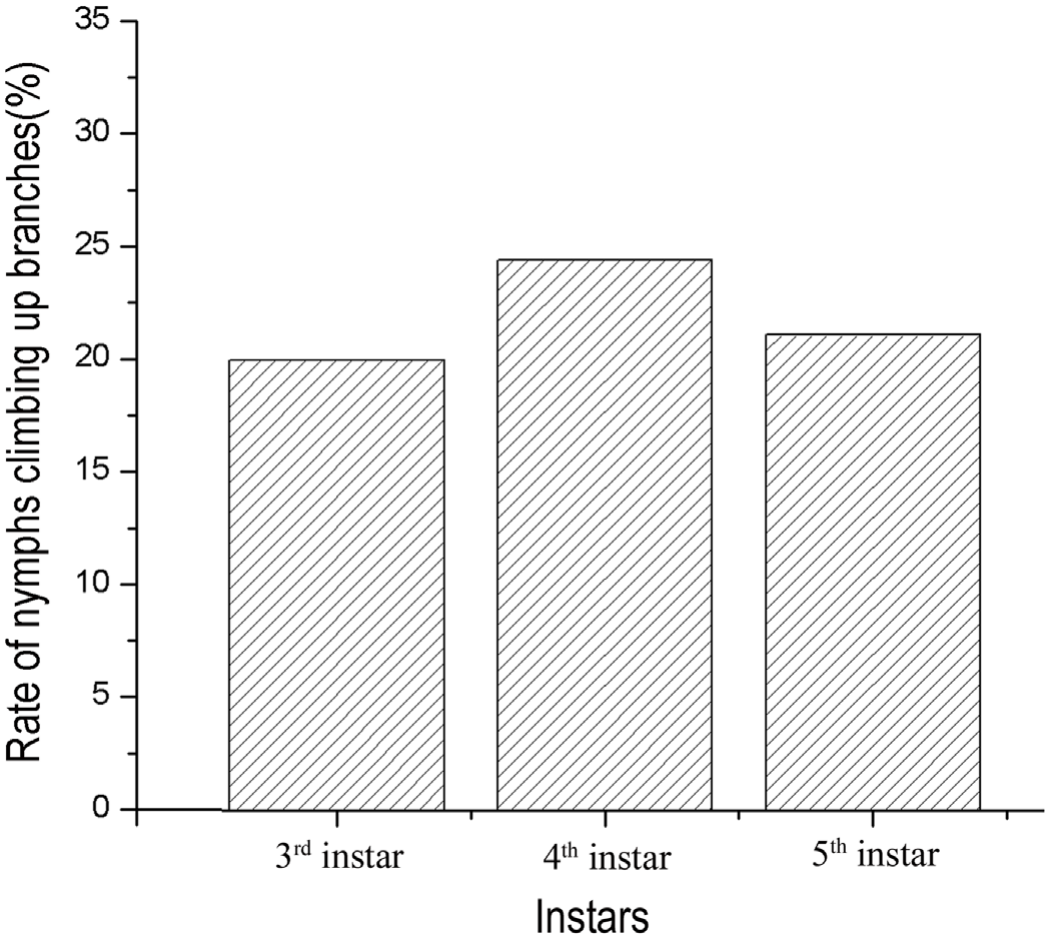
Rates of Different Nymph Instars Climbing Branches of *P*. *acerifolia*.

The *t-test* for independent samples showed that *C*. *ciliata* nymphs had significantly different location capabilities for host and non-host trees (*t* = 5.82, *P*<0.01). Approximately 21.85% of the tested nymphs climbed up the *P*. *acerifolia* branches with 3–5 leaves in four directions within 24 hours. By contrast, only 1.85% of the tested nymphs climbed the control (non-host: *Populus canadensis*) branches (Fig 5). Multiple comparisons revealed that there were no significant differences between the different instars in their ability to locate host trees. The rates at which the tested nymphs climbed up the branches of host trees during the 3^rd^, 4^th^, and 5^th^ instars were 20.00%, 24.44% and 21.11%, respectively (Fig 6).

### Host location of *C*. *ciliata* adults

At the second day after their release 2 m away from the forest edge, 20 adults were captured in the forest plot, accounting for 20% of the total released adults. Their average flight distance was 3.55 m, and the maximum distance was 6 m. When they were released 10 m away from the forest edge, 13% of the released adults could fly to the forest within one day. They flew an average of 12.96 m, with the longest distance being 19 m. Eleven adults were found in the forest at one day after the release at 20 m away from the forest edge; their average flight distance was 22.14 m, and one was captured 27 m away from the release point, which was the longest distance in that group. No adults were captured in the jujube, poplar and elm trees that were 2–8 m away from the release points (Table 2).

**Table 2:** Flying Distance of Captured Adults from Three Release Points.

Release points	CAP			CAO
	Number	Flying distance (m, Mean±SE)	Maximum flight distance (m)	Number
2 m	20	3.55±0.26	6	0
10 m	13	12.96±0.78	19	0
20 m	11	22.14±0.95	27	0

CAP, Captured adults in *P*. *acerifolia* forest; CAO, Captured adults in jujube, poplar and elm trees.

## Discussion

The speed and distance of insect movement have been the focus in the vast majority of studies, whether of pests or natural enemies [20,21,43–47], and they are affected by both intrinsic (age, life cycle stage, gender, and physical condition) factors and extrinsic (conditions, resources, interactions with other organisms, and barriers to movement) factors [47]. Results from this study indicate that the moving speed increased with the age of *C*. *ciliata* nymphs, and similar results were obtained for the crawling distance as tested in the laboratory (Table 1). Fifth-instar nymphs moved an average of 0.038 m/min during the first 15 minutes after release, which was much faster than the average moving speed of the larvae of *Altica subplicata* LeC. (43 cm/day, which is equivalent to 0.0003 m/min) [41]. *C*. *ciliata* nymphs could move as far as 750 cm throughout their own lifespan, demonstrating that the nymphs also had strong diffusion potential in the forest. Although there were marked differences in crawling time between the different ages of *C*. *ciliata* nymphs tested (Table 1), the difference in crawling distance per unit time was not significant. This indicates that the older nymphs may have greater endurance than younger nymph, but the mechanism underlying this phenomenon requires further explanation.

Insects must try their best to search for, identify and locate new hosts as soon as possible to obtain essential nutrients for their growth and development once they are forced to leave their original hosts. Many studies have shown that plant volatiles are the main cues used by phytophagous insects in orientation to their host plants [48–54], and the visual cues such as plant shape and color also played an important role in the process [55–59]. The current study shows that 21.85% of the tested nymphs climbed the four *P*. *acerifolia* branches within 24 hours, whereas only 1.85% of the nymphs climbed the control branches (non-host: *Populus canadensis*) (Fig 5), which means that there may be not visual but chemical orientation toward host plants for *C*. *ciliata* nymphs. Some studies suggested that lavae ages also had influence on host location ability [59–61]. But this article showed different results that the tropism is not different between different instars of nymphs based on their rate of climbing the branches of host trees (3^rd^ instar: 20.00%; 4^th^ instar: 24.44%; 5^th^ instar: 21.11%) for *C*. *ciliata* (Fig 6). The mechanism needs further study.

The flying wings of adults of *C*. *ciliata* are very delicate, and these insects therefore rarely fly very far [62]; however, Maceljski [63] writes that adults "are very mobile and are good fliers". Actual measurements of flying distance have been made in this paper and their maximum flying distance tested in one day was 27 m (Table 2). Due to limited space, adults of *C*. *ciliata* were not released at a further distance, and they may fly longer distances. However, the longest flying distance in one day (27 m) showed that the opinion from Maceljski [63] is correct. Not only that, *C*. *ciliata* adults could fly faster than many insect species that are larger or smaller than them, such as *Lygus hesperus* Knight (4.6 m/d), *Anoplophora glabripennis* Motchulsky (20 m/d) and *Diaphorina citri* Kuwayama (5.8 m/d) [21,44,64].

Mark-release-recapture methods are a very common method of estimating insect movement in many experiments [5,65–71]. The advantage of these methods is that they allow accurate characterization of dispersal behavior in the natural environment [72], but several key technical problems need to be solved. First, a long-term non-invasive marker should be selected for each insect species. The need to mark individual insects limits the size of the population that can be marked, reducing the likelihood of detecting rare long-distance dispersal events [73]. Second, the appropriate number of released insects to enable adequate recapture frequencies for statistical analyses [74] may result in density-dependent movement, leading to biased estimates of movement rate [5]. In addition, another critical problem may be the recapture method. For example, highly efficient attractants are often used in the re-identification of released adult populations. The observed dispersal distance would become shorter because the attractant shortens the dispersal distance by intercepting individuals who should have dispersed for longer distances [27]. Even if the problem above is solved, mark-release-recapture experiments have been limited by being conducted at relatively small scales [73], and predictions of movement only indicate how populations will respond in similar circumstances [75]. It must be clearly recognized that obtaining unbiased estimates of the distribution of animal dispersal distances in natural unbounded populations has long been known to be problematic [4]. Although following movement in the field is very difficult for small insects [76], some behaviors of both nymphs and adults of *C*. *ciliata* in this article were visually observed throughout the process of the release-recapture method, without disturbance from marking and luring that may affect movement behavior.

In short, results from this study indicated that both the nymphs and adults of the sycamore lace bug, *C*. *ciliata*, have some active diffusion capacity, which may very helpful in the practice of prevention of *C*. *ciliata*. For instance, based on the maximum distance that the nymphs crawl all the host need spraying at a distance of 750cm where the leaves are infesting by nymphs. Similarly, the scope of prevention needs to expand to at least 27m if the harm was caused by adults.
